# Early Detection of Cervical Cancer by Fluorescence Lifetime Imaging Microscopy Combined with Unsupervised Machine Learning

**DOI:** 10.3390/ijms231911476

**Published:** 2022-09-29

**Authors:** Mingmei Ji, Jiahui Zhong, Runzhe Xue, Wenhua Su, Yawei Kong, Yiyan Fei, Jiong Ma, Yulan Wang, Lan Mi

**Affiliations:** 1 Department of Optical Science and Engineering, Shanghai Engineering Research Center of Ultra-Precision Optical Manufacturing, Key Laboratory of Micro and Nano Photonic Structures (Ministry of Education), School of Information Science and Technology, Fudan University, 220 Handan Road, Shanghai 200433, China; 2 Institute of Biomedical Engineering and Technology, Academy for Engineering and Technology, Fudan University, Shanghai 200433, China; 3 Shanghai Engineering Research Center of Industrial Microorganisms, The Multiscale Research Institute of Complex Systems (MRICS), School of Life Sciences, Fudan University, 220 Handan Road, Shanghai 200433, China; 4 Department of Gynecology and Obstetrics, The Central Hospital of Wuhan, Tongji Medical College, Huazhong University of Science and Technology, 26 Shengli Street, Wuhan 430014, China

**Keywords:** cervical cancer, non-invasive screening, NAD(P)H, fluorescence lifetime imaging microscopy, unsupervised machine learning

## Abstract

Cervical cancer has high morbidity and mortality rates, affecting hundreds of thousands of women worldwide and requiring more accurate screening for early intervention and follow-up treatment. Cytology is the current dominant clinical screening approach, and though it has been used for decades, it has unsatisfactory sensitivity and specificity. In this work, fluorescence lifetime imaging microscopy (FLIM) was used for the imaging of exfoliated cervical cells in which an endogenous coenzyme involved in metabolism, namely, reduced nicotinamide adenine dinucleotide (phosphate) [NAD(P)H], was detected to evaluate the metabolic status of cells. FLIM images from 71 participants were analyzed by the unsupervised machine learning method to build a prediction model for cervical cancer risk. The FLIM method combined with unsupervised machine learning (FLIM-ML) had a sensitivity and specificity of 90.9% and 100%, respectively, significantly higher than those of the cytology approach. One cancer recurrence case was predicted as high-risk several months earlier using this method as compared to using current clinical methods, implying that FLIM-ML may be very helpful for follow-up cancer care. This study illustrates the clinical applicability of FLIM-ML as a detection method for cervical cancer screening and a convenient tool for follow-up cancer care.

## 1. Introduction

Cervical cancer is one of the top four cancers that affect women’s life and health, with approximately 600,000 new cases each year worldwide [1]. Compared with developed countries, developing countries have much higher morbidity and mortality rates [2,3]. Cervical cancer screening usually includes human papillomavirus (HPV) DNA testing, cytology, or a combination of the two tests. Although cervical cancer screening can reduce mortality to an extent, there are limitations. For example, HPV positive does not necessarily mean that the patient needs intervention and treatment, and the sensitivity and specificity of the cytology test are unsatisfactory. The relatively low sensitivity of liquid-based cytology (LBC) screening has also been reported in different studies, ranging from 52% to 94% [4], and the specificity may be as low as 73% [5]. After cervical cytology, patients with low- or high-grade squamous intraepithelial lesions (LSILs or HSILs) should undergo colposcopy and histopathological biopsy. Patients with atypical squamous cells of uncertain significance will be recommended a repeat cytology test in a close follow-up visit, or colposcopy and histopathological biopsy. A biopsy is an invasive procedure that can lead to bleeding, infection, and physical and psychological suffering. Therefore, a screening method with high sensitivity and specificity is needed to assist cervical cancer screening and reduce false positives (FPs).

Fluorescence lifetime imaging microscopy (FLIM) has received increasing attention in the biomedical field as a label-free and highly sensitive optical detection technology [6,7,8,9]. It is well known that during the development of cancer, the metabolism of cancer cells changes substantially compared with normal cells and can be detected by an endogenous coenzyme involved in biological metabolism, namely, reduced nicotinamide adenine dinucleotide (phosphate) [NAD(P)H] [10]. Thus, we can use FLIM to detect the fluorescence lifetime of intracellular NAD(P)H and evaluate the metabolic status of cells or tissues. Recently, there have been a series of reports on the study of unstained cervical tissue sections by FLIM, and it was found that FLIM has application potential in cervical cancer detection [11,12,13]. However, the samples in the abovementioned reports were all tissue sections and the samples were invasively obtained from biopsies or surgeries, which is not practical for widespread screening. As the method of obtaining exfoliated cervical cells is non-invasive, the exfoliated cells can be used as FLIM samples instead of tissue samples.

In the past, FLIM image analysis mainly extracted the lifetime value manually [8,9,14], which is inefficient when a large amount of sample data is involved. In addition, when there is a large difference among different cells in the same case or when the cancer is in the early stage, the fluorescence lifetime value distribution may be large. As a result, the difference in the average value between the cancer group and the normal group may be small, and the classification accuracy may thus be limited. Machine learning (ML) has helped further the development of medical image classification and quantification. Considering the expensive hardware of FLIM, Mannam et al. trained a neural network model to estimate FLIM images from conventional fluorescence intensity images of a zebrafish labeled with enhanced green fluorescence protein by a two-photon microscope [15]. However, this model has limitations, as the authors stated that the model behaves differently for stained and unstained samples, and the training dataset needs a large amount of lifetime and intensity image pairs for each fluorescent molecule. ML was reported suitable for the analysis and interpretation of FLIM raw data (either in time-domain or frequency-domain) [15]. Gang et al. used an artificial neural network (ANN) method to estimate the lifetime from the FLIM raw data, and found the method was more accurate and faster compared to curve fitting tools [16]. A convolutional neural network (CNN) was also used to extract the lifetime from raw data and reconstruct the FLIM images [17]. After the FLIM images are obtained, ML can be used in applications such as segmentation [18] and classification [19,20,21]. The application of classification in cells or tissues using FLIM combined with ML is attractive. The wide applications include tumor biomarker analysis [22,23,24], embryo quality estimation for in vitro fertilization [19], microglia recognition from other glia cell types in the brain [20], and the hematoxylin and eosin-stained cervical tissue study for precancer detection [21]. Most of the reports, however, used supervised learning [19,20,21].

ML is mainly divided into two categories: supervised learning and unsupervised learning [25,26]. Supervised learning requires that the input data have a clear category label and that the algorithm can find a mapping relationship with the target category from a large amount of training data. Unsupervised learning, unlike supervised learning, does not have explicit class labels for the input data during the learning process. For example, in the field of medical diagnosis, it is very time-consuming to have pathologists label hundreds or thousands of images one by one. Therefore, unsupervised learning is of great practical significance, and such algorithms may help discover relationships in unlabeled data. With the help of high-performance algorithms, multi-dimensional information can be integrated for the automatic feature extraction and classification of image data.

Regarding cervical cytology, many researchers have carried out supervised learning on microscopic images of stained cells to classify normal and abnormal cells. Most classification results with high accuracy were obtained on single-cell images [27,28,29] because only non-overlapping cells can help distinguish the nuclear area easily when stained cells are involved. However, exfoliated cervical cells tend to overlap and aggregate, and the method of observing cell morphology by stained cells will encounter difficulties in clinical application. When observing overlapping cells or aggregated cells, FLIM images can provide information on the metabolic status even if the determination of cell morphology is affected, which is more suitable for practical applications. Therefore, the present work used FLIM technology to observe unstained exfoliated cervical cells combined with unsupervised machine learning to analyze the FLIM images of cervical cells. This FLIM-ML method does not need to label cells, which greatly enhances and improves the efficiency, specificity, and sensitivity of cervical cancer screening, and thus provides a new method for the early screening of cervical cancer or follow-up examinations after cancer treatment.

## 2. Results and Discussion

### 2.1. NAD(P)H FLIM Images of Exfoliated Cervical Cells

**Figure 1:**
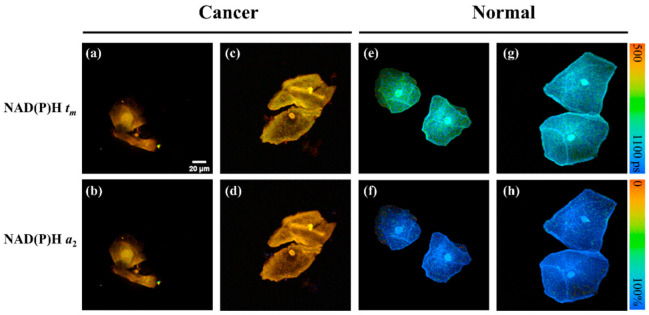
Typical FLIM images of unstained exfoliated cervical cells from four participants (each column is from one person); (**a**–**d**) are from two cervical cancer patients and (**e**–**h**) are from two normal cases where the autofluorescence is from the intracellular NAD(P)H; *t_m_* means the mean fluorescence lifetime of NAD(P)H; and *a*_2_ means the contribution of protein-bound NAD(P)H. Scale bar: 20 µm.

The 71 participants in this study were divided into several groups depending on their clinical diagnosis: cervical cancer (CC, n = 11), cervical intraepithelial neoplasia grade 2/3 (CINII/III, both CINII and CINIII are considered HSILs, n = 7), benign (n = 18), normal (n = 23), and follow-up (n = 12). It should be noted that LSILs are not involved in this study because the recommendations for LSILs are to avoid treatment and continue monitoring. Figure 1 shows the typical FLIM images of unstained exfoliated cervical cells from two cervical cancer patients (Figure 1a–d) and two normal cases (Figure 1e–h) where the autofluorescence is from the intracellular NAD(P)H; *t_m_* means the mean fluorescence lifetime of NAD(P)H; and *a*_2_ means the contribution of protein-bound NAD(P)H. As can be seen from the cell morphology, the cell nuclei in Figure 1a,b are abnormally enlarged and have a relatively large nuclear-cytoplasmic (NC) ratio, which is typical of cancer cell characteristics, compared to the other cell images. However, the cytological morphology in Figure 1c,d seems normal. This suggests that not all cells from cancer patients display cancer cell cytological characteristics, and such normal-like cells may cause misdiagnosis if examined by the LBC test.

In addition to displaying cell morphology, FLIM can also provide information about the fluorescence lifetime. As shown in the right bar of Figure 1, for *t_m_*, orange indicates a short fluorescence lifetime and blue indicates a long lifetime; for *a*_2_, orange indicates a low ratio of protein-bound NAD(P)H and blue indicates a high ratio of protein-bound NAD(P)H. As presented in the typical FLIM images, cancer cells have a relatively short average fluorescence lifetime (*t_m_*) and less protein-bound NAD(P)H ratio (*a*_2_). This implies that, compared with normal cells, cancer cells favor glycolysis rather than oxidative phosphorylation. This result is consistent with numerous previous reports regarding the Warburg effect [30,31,32]. Although the cells in Figure 1c,d are morphologically normal, their FLIM images are significantly different from those of normal cells, which show a yellow color. This may be explained by the fact that the cytological morphology of some cells has not changed, but their abnormal metabolic status can already be sensitively detected by FLIM.

This work studied thousands of FLIM images taken from real clinical samples. Image preprocessing was necessary and involved data filtering to ensure the validity of the analyzed data. Qualified images demonstrating cervical cells (as shown in Figure S1a) were chosen for further study, and some images in which the fluorescence of cervical cells was not severely affected by surrounding objects were also qualified. Figure S1b,c shows examples of unqualified images. In Figure S1b, the fluorescence intensity of the non-cellular area is much stronger than the cellular area, which would lead to inaccurate data fitting. In Figure S1c, many neutrophils cover the exfoliated cervical cells, which would result in the fluorescence information being biased and thus affecting the subsequent analysis. In addition, excessive numbers of neutrophils, erythrocytes, or microorganisms may affect the image quality, thus data filtering is required. Therefore, images such as Figure S1b or Figure S1c should be filtered out, and qualified FLIM images can be selected for subsequent analysis.

If the noise of FLIM images is reduced, the accuracy of classification can be increased. Mannam et al. performed fluorescence intensity denoising using ‘Noise2Noise’ CNN for the mixture of Poisson–Gaussian noise [33] and the same Noise2Noise pre-trained model to denoise FLIM images [15]. The reported method is of high accuracy [15,33] and may improve the accuracy of classification, but a large number of raw images are needed for neural network training. Acquiring 12,000 real fluorescence microscopy images and 60,000 noisy images with different noise levels [33] is time-consuming. To reduce the image noise in the present work, data filtering for qualified images and smoothing images with 3 × 3 spatial filtering were performed, which is computationally efficient.

### 2.2. Statistical Analysis of FLIM Images and Dataset Selection

**Figure 2:**
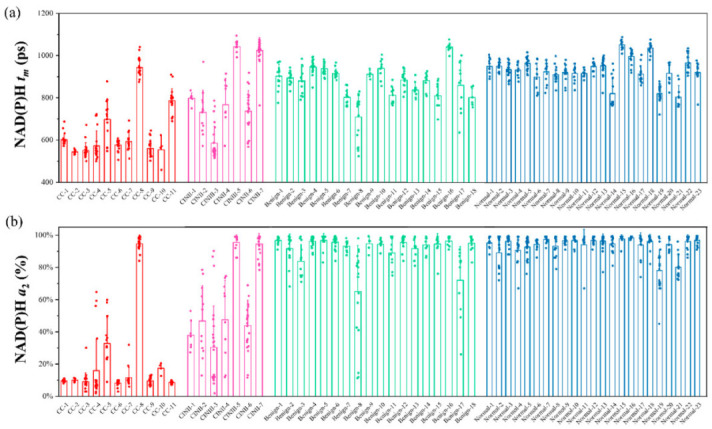
Statistical FLIM data of exfoliated cervical cells from the CC (n = 11), CINII/III (n = 7), benign (n = 18), and normal (n = 23) groups. (**a**) The average fluorescence lifetime (*t_m_*) of NAD(P)H of cervical cells based on the peak values of the FLIM distribution curves. (**b**) The protein-bound NAD(P)H proportion (*a*_2_) of cervical cells based on the peak values of the FLIM distribution curves. Each column represents one participant, and each circle represents one FLIM image data.

Each pixel in the FLIM image corresponds to a *t_m_* value and an *a*_2_ value; thus, each 256 × 256 pixel image has two distribution curves of *t_m_* and *a*_2_. The peaks of the distribution curves were used for statistics as presented in Figure 2, in which the *t_m_* and *a*_2_ data were from CC, CINII/III, benign, and normal groups. Each column represents one participant, and each circle represents one FLIM image data. The average *t_m_* of the above four groups was 647 ± 137 ps, 805 ± 187 ps, 878 ± 91 ps, and 928 ± 70 ps, respectively, and the average *a*_2_ was 21.7 ± 28.1%, 57.8 ± 31.4%, 90.6 ± 13.8%, and 93.4 ± 8.2%, respectively. It can be found that there is little difference in metabolism between the normal group and the benign group, suggesting that the benign group has a similar metabolic state as the normal group. The cancer group had the lowest *t_m_* and *a*_2_ values, and the CINII/III group was between the normal and cancer groups. These statistical results show a similar trend in Figure 1, indicating that metabolic state changes are prevalent in cancer and CINII/III cases.

Although there was some difference in the average values of the four groups, the individual difference in the same group was large, i.e., there were large differences in different cells from the same patient for the CC, CINII/III, and benign groups especially. Therefore, it is difficult to classify each patient by directly setting the threshold of the *t_m_* and *a*_2_ values. Additionally, extraction of the *t_m_* and *a*_2_ values from the FLIM images using exponential fitting software is time-consuming and labor-intensive. To solve these problems, this study combined FLIM with an unsupervised algorithm to quantitatively predict the cancer risk for each patient.

**Figure 3:**
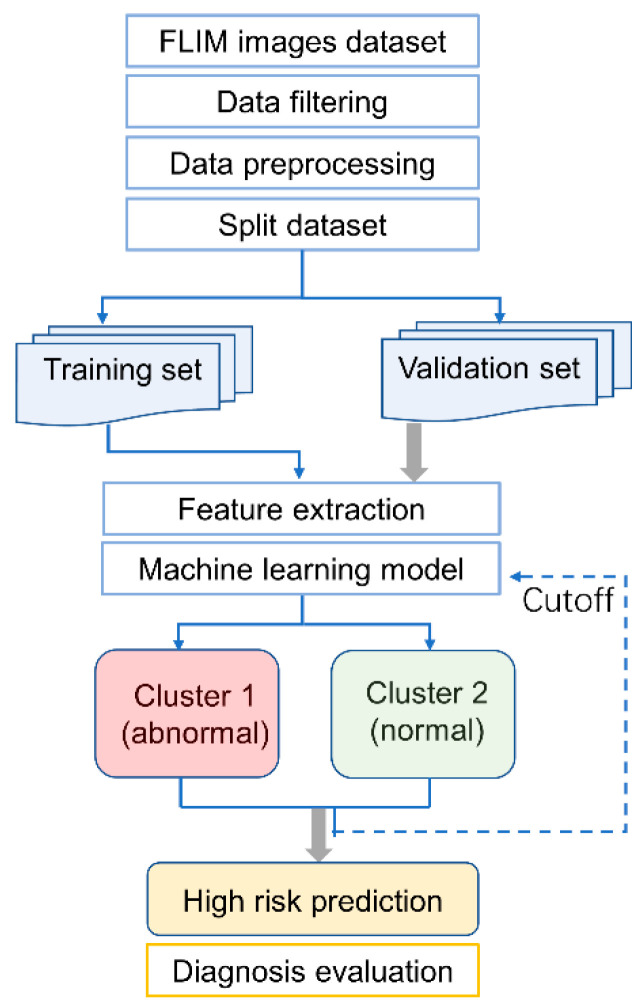
Flow chart of the FLIM-ML model for the prediction of high risk of cervical cancer.

For a cervical cancer case, the exfoliated cervical cell sample may contain thousands of cells. It is possible that not all cells are malignant, especially in patients with early-stage cancer or HSIL. Therefore, it is difficult to label all cell images accurately. To obtain a reliable training model, 151 images from 5 patients with cervical cancer and 4 patients with CINII/III, and 217 images from 14 women of the normal group whose LBC test, HPV test, and ultrasound report were all negative, were selected as the training dataset. The other 48 participants were designated as the validation dataset. The distribution of the participants is listed in Table 1. The flow chart of the FLIM-ML model for the prediction of high risk of cervical cancer is presented in Figure 3.

### 2.3. Result of Feature Extraction and PCA

To improve the efficiency of image analysis, cell region segmentation and cell mask image acquisition were performed as shown in Figure S2 (the details are described in the Materials and Methods section). Then, the AlexNet network, which was pre-trained on the ImageNet database, was used to extract feature descriptors of each image. Finally, 9216 features were extracted. Next, principal component analysis (PCA) [34] was applied to reduce the dimensionality of the data. The distribution of high-dimensional features can be visualized by t-distributed stochastic neighbor embedding (t-SNE) [35].

**Figure 4:**
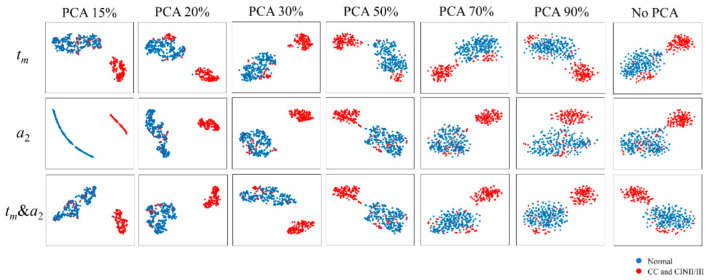
t-SNE projection of feature data extracted from three input images of the training dataset and the preserved different total variances of the data. Each point represents one FLIM image data. Blue points are from 217 FLIM images of the normal group and red points are from 151 FLIM images of cervical cancer or CINII/III groups.

There were three kinds of FLIM images as input: *t_m_* images, *a_2_* images, and *t_m_* & *a*_2_ images in the same field of view. *t_m_* or *a*_2_ images were RGB images containing three channels. For the *t_m_* & *a*_2_ images, each image contained six channels. Figure 4 shows the t-SNE projection of feature data extracted from three input images of the training dataset using the pre-trained network. Each point represents one FLIM image. Blue points are from 217 FLIM images of the normal group and red points are from 151 FLIM images of cervical cancer or CINII/III groups. Of the total variance of the data, 15%, 20%, 30%, 50%, 70%, and 90% were preserved after PCA. It can be seen that when 15% was preserved, the t-SNE projection of feature data extracted from the *a*_2_ images was questionable, which might be due to the loss of original information. The distance of data between the two clusters was far when 20% or 30% of the total variance of data was kept, indicating that the visual differences between the images of the two groups were generally consistent with the result shown in Figure 2. With the preserved variance increasing from 30% to 90%, the distance between the two clusters became progressively closer. The extracted features were more dispersed in the same group without PCA, indicating that the output features of the original data contained noise that drowned out the useful signal. When comparing 20% and 30% of the total variance, the lower variance corresponding to lesser information may lead to a lower classification performance. Therefore, components that preserved 30% of the variance in the original data can benefit the cluster, thereby improving the classification performance and reducing the computational cost.

### 2.4. Results of Clustering and the FLIM-ML Model

The k-means algorithm was used for clustering after feature extraction. Table 2 lists the clustering results of the 368 images in the training dataset. The images from the CC/CINII-III and normal groups were classified into two clusters, and the results obtained from the three different sets of input images were essentially consistent. According to the results, the majority of *t_m_* images from the normal group were classified in cluster 2 and all of the *a*_2_ images and *t_m_* & *a*_2_ images from the normal group were also classified in cluster 2. Thus, cluster 2 should be defined as normal. For the images from CC/CINII-III, 75.5% of images were classified in cluster 1. It can be understood that not all cells from CC/CINII-III are malignant, especially in early-stage patients, therefore, cluster 1 was defined as abnormal. Subsequently, the model built on the training dataset was applied to the validation dataset for diagnostic evaluation.

The validation dataset consisting of 48 participants from five groups based on their medical history and clinical diagnosis, CC (n = 6), CINII/III (n = 3), benign (n = 18), normal (n = 9), and follow-up (n = 12), is listed in Table 3. The follow-up group consisted of follow-up patients after the surgical treatment of cervical cancer, and two of them were found to have vulvar or vaginal intraepithelial neoplasia (VIN or VaIN). Table 3 lists the percentage of abnormal images from each participant in the validation dataset. Among the 6 CC patients, 5/6 of them were predicted to be abnormal and almost 100% of images were abnormal. For the three CINII/III patients and the two cancer recurrence patients, the percentage of abnormal cell images was not 100%, which is consistent with the speculation that not all cells were malignant, especially in an early stage. A few benign, normal, and follow-up patients had some abnormal cells. Therefore, the cutoff percentage that may imply high risk needs to be calculated for the FLIM-ML model.

**Figure 5:**
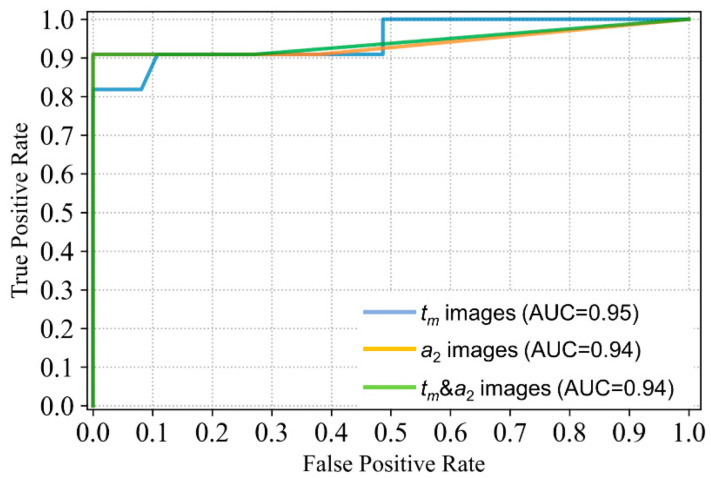
ROC curve and AUC for the three different input images.

To evaluate the model performance, receiver operating characteristic (ROC) curves were plotted, and the area under the curve (AUC) was calculated. As shown in Figure 5, the AUC with different input images were 0.95, 0.94, and 0.94, respectively. Based on the ROC, the optimal cutoff value for the percentage of abnormal images was determined by maximizing the Youden index (maximum sensitivity plus specificity minus 1). For different input images, the cutoff values were 74%, 68%, and 45% for *t_m_* images, *a*_2_ images, and *t_m_* & *a*_2_ images, respectively. In this study, if the percentage of abnormal images for one patient exceeded the cutoff value, it was determined to be positive (malignant); it was deemed negative (normal or benign) if it was less than the cutoff. It was found that the sensitivity of FLIM-ML based on *t_m_* input images was lower than those based on *a*_2_ images and *t_m_* & *a*_2_ images. Therefore, the FLIM-ML prediction results (+ or −) listed in Table 3 were obtained by comparing the abnormal percentage with 68% for *a*_2_ images and 45% for *t_m_* & *a*_2_ images. The best result was obtained with *t_m_* & *a*_2_ images as input, which implies that the six-channel images composed of *t_m_* and *a*_2_ contain more useful information. This result agrees well with several works of literature reporting that the classification results using combined features were better than using one type of feature [36,37].

### 2.5. Results of FLIM-ML and Its Comparison with LBC

**Figure 6:**
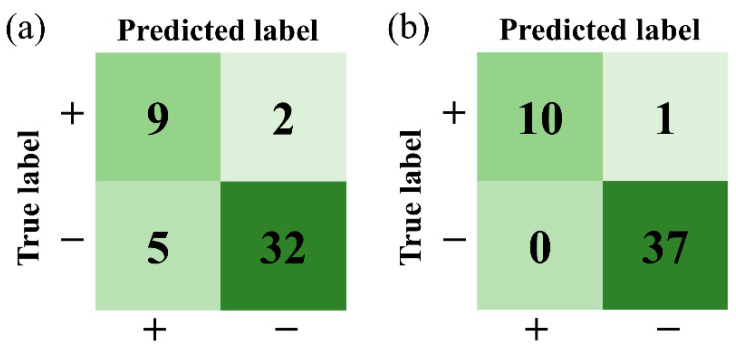
Confusion matrixes of the two methods: (**a**) LBC test; (**b**) FLIM-ML method.

Clinical diagnosis based on colposcopy examinations, clinical laboratory tests, ultrasound, and histopathology examinations was made by physicians and was considered the standard reference. According to the standard, the FPs and false negatives (FNs) of the FLIM-ML method and LBC test were evaluated and are marked in Table 3. For all patients with CC, CINII/III, VIN, and VaIN, the prediction of positive is true positive (TP). For all other participants, namely, benign, normal, and follow-up without detectable new lesions, the prediction of negative is true negative (TN). Confusion matrixes are presented in Figure 6 to visualize the performance of the LBC test and the FLIM-ML method for the validation dataset containing 48 participants. The LBC tests reported two FNs and five FPs. The FLIM-ML method reported one FN and no FPs. The sensitivity and specificity of the two methods were quantified and are listed in Table 4. The FLIM-ML method exhibits a good potential for reducing FPs, thereby potentially reducing unnecessary biopsies. Additionally, the FLIM-ML method showed a higher sensitivity than the LBC method in this study.

**Figure 7:**
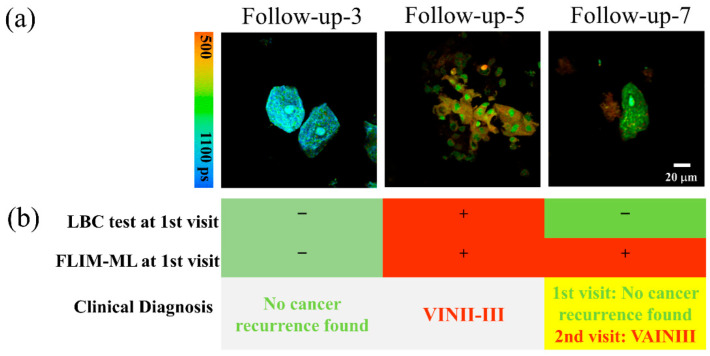
Results of three follow-up patients. (**a**) FLIM *t_m_* images of the three patients. LBC test; (**b**) the results of the LBC test and FLIM-ML method and the clinical diagnosis. Follow-up-7 was predicted as high risk by FLIM-ML at the first follow-up visit but was judged normal by the current clinical methods. The cancer recurrence of Follow-up-7 was not clinically found until the second visit eight months later.

There were some important results found for the patients in the follow-up group. Figure 7 shows the typical FLIM *t_m_* images from three patients. Follow-up-3 was a patient without detectable cancer recurrence at her follow-up visit. Follow-up-5 was diagnosed with VINII-III one year after cervical cancer surgery, with cell morphological features and FLIM *t_m_* values significantly different from those of Follow-up-3. For the above two cases, the results of LBC and FLIM-ML were consistent. However, FLIM-ML showed advantages for Follow-up-7. At the first visit of this patient, the LBC test only reported inflammatory cells (meaning negative); the HPV test reported 16+ and 53+ and the biopsy showed no abnormality. However, after examining the spare LBC liquid by the FLIM-ML method, the percentage of abnormal cells was found to be 76%, so Follow-up-7 was predicted high risk. It can also be seen from Figure 7a that there was no obvious abnormality in the cell morphology of Follow-up-7, which may explain the negative LBC result at the first visit. The patient (Follow-up-7) was reexamined for the second time eight months later, and the pathology report was VAINIII. This case suggests that the FLIM-ML method may predict high risk before abnormal cytology, which may be very helpful for follow-up cancer care, early detection, and treatment. Further study on follow-up cases is thus necessary.

## 3. Materials and Methods

### 3.1. Participants and Exfoliated Cervical Cell Samples

The study was approved by the Institutional Ethics Committee of the Central Hospital of Wuhan, Tongji Medical College, Huazhong University of Science and Technology, China. The work involved 71 women with an average age of 43 years who had a definite diagnosis. The clinical diagnosis made by doctors based on colposcopy examinations, clinical laboratory tests, ultrasound, and histopathology examinations was set as the standard reference. For the CC group (n = 11) and the CINII/III group (n = 7), no prior diagnosis of cervical cancer or precancerous lesions was found before the present examination. For the benign group (n = 18) and the normal group (n = 23), the LBC test, HPV test, and ultrasound examination had been performed, and some of them with LBC and/or HPV positive results underwent biopsies due to clinical recommendations while no malignant sign was detected. For the follow-up group (n = 12), cervical cancer or CINII/III had been diagnosed, and gynecological surgeries had been performed from ten months to four years prior. It should be noted that CINI (LSILs) were not studied in this work because the recommendations for LSILs are to avoid treatment and continue to monitor.

The exfoliated cervical cell samples were obtained from the spare LBC test liquid after the routine cervical cytology test by the department of pathology. After the LBC tests, the remaining LBC liquid containing exfoliated cells was centrifuged at 1000 rpm for 3–5 min. P of the supernatant was discarded, and the bottom sediment was mixed with the remaining liquid. A few drops of liquid were dripped on a clean glass side and covered with a coverslip for FLIM observation. It should be noted that the only difference between the cell samples of this study and the LBC test was that the LBC test requires staining and the FLIM method studies unstained cells.

### 3.2. Fluorescence Lifetime Imaging and Analysis

The fluorescence lifetime images were acquired by a time-correlated single-photon counting system (SPC-150, Becker & Hickl, Berlin, Germany) on a laser scanning confocal microscope (FV300/IX 71, Olympus, Tokyo, Japan) with a water-immersion objective lens (60×, NA = 1.2, Olympus, Tokyo, Japan). The samples were excited by a 405 nm picosecond laser (50 MHz, BDL-405-SMC, Becker & Hickl, Berlin, Germany) and collected by a photomultiplier tube (PMC-100-1, Becker & Hickl, Berlin, Germany) with a 447 ± 30 nm bandpass. Each FLIM image of 256 × 256 pixels was acquired in 20–60 s, and an area with the size of approximately 188 × 188 μm was imaged only once to avoid photobleaching. At least ten different areas were imaged for each sample, and 15–50 cells were observed for each patient.

FLIM images were fitted with double-exponential decay models using the commercial SPCImage software (SPCImage v.8.0, Becker & Hickl, Berlin, Germany). The mean lifetime of each pixel *t_m_* can be obtained by the following formula:

(1) $$t_{m}=a_{1}\times t_{1}+a_{2}\times t_{2},$$

where $a_{1}$ and $a_{2}$ are the contributions of free and protein-bound NAD(P)H and $t_{1}$ and $t_{2}$ are the fluorescence lifetimes of free and protein-bound NAD(P)H, respectively. In this study, $t_{1}$ was fixed at 460 ps, according to the experimental result of free NADH solution measured by the FLIM setup. Then, the $t_{2}$, $a_{2}$, or $t_{m}$ value of all pixels in each FLIM image could be obtained using SPCImage software. In this study, $t_{m}$,$\;a_{1}$, $a_{2}$, $t_{1}$, and $t_{2}$ are five parameters that can be extracted from the FLIM raw data. Since $a_{1}$ = 1 − $a_{2}$ and $t_{1}$ was fixed as the fast decay component from free NADH, only three parameters ($t_{m}$, $a_{2}$, and $t_{2}$) could be studied. It should be noted that the assumption that the NAD(P)H has two decay components is a simplification. There is evidence that the slow decay component ($t_{2}$) consists of at least two or three subcomponents [38,39]. In addition, $t_{m}$ and $a_{2}$ (or the $a_{1}$/$a_{2}$ ratio) has been used to study metabolism in numerous pieces of literature [6,11,12,13,20,21,29]. Therefore, $t_{m}$ and $a_{2}$ were studied in this work.

### 3.3. FLIM Images Preprocessing

After data filtering as mentioned in Section 2.1 and Figure S1, all qualified FLIM images were further preprocessed by segmentation. An Otsu-based automatic segmentation algorithm was applied. The implemented algorithm is schematically shown in Figure S2. First, Otsu’s thresholding-based method was used to separate the pixels into the background and foreground by finding the optimal threshold for segmenting an image. Second, a morphological dilate operation was performed on the binary image with a structuring element, in which a circle with a radius of 1 pixel worked the best. Next, to remove the non-cellular regions, the connected components were then found in the binary image, and we filtered the areas with small pixels. Finally, any holes in this region were filled, and the image was smoothed to remove a small amount of noise using 3 × 3 spatial filtering. The final cell mask images were used to extract the image features.

### 3.4. Unsupervised Machine Learning Method

The AlexNet network [40,41] developed by Krizhevsky achieved the top score in the ImageNet Large Scale Visual Recognition Challenge 2012. It is a popular convolutional neural network for computer vision tasks because of its high performance and relative simplicity [42]. AlexNet, which was pre-trained on the ImageNet database, was used to extract the feature descriptors of each image, and 9216 features were finally extracted. These outputs included a significant amount of noise and zero elements resulting from filters that have not been activated. To increase the classification performance and decrease the computational cost simultaneously, PCA was applied to reduce the dimensionality of the data. This method converted multiple variables into a few principal components that reflected most of the information of the original variables. The principal components are not mutually related, which ensures that the information contained in the principal component does not overlap. A popular method, t-SNE, was used to project high-dimension data into two dimensions so that it could be analyzed visually. The distance between the points is likely to be representative of the actual distances in the original feature space.

Since every sample contains hundreds or thousands of cells and there are likely normal cells in the samples for some cancer patients, it would be impossible to give a definite label to every cell without doctors’ help. Even if doctors can accurately label each cell, the workload is huge, and the doctors’ labeling may be affected by multiple factors, such as sampling, experience, and image quality. Given this situation, clustering was used in this study to assign labels to images and group nearby points in the feature space. K-means is an unsupervised machine learning method and one of the most popular clustering algorithms. In the algorithm, K is the number of clusters, which was set to two for the training dataset based on the clinical diagnosis. When selecting the starting centroids in the algorithm, a method called k-means++ [43] is used to help k-means achieve good clustering performance and computational efficiency.

## 4. Conclusions

In this work, exfoliated cervical cell samples from 71 women were collected and the autofluorescence of the cell samples was observed using FLIM. It was found that cancer cells and normal cells had significant differences, suggesting that cancer cells favor glycolysis rather than oxidative phosphorylation. FLIM images were studied by the unsupervised machine learning method to predict the cancer risk for patients. The sensitivity and specificity of the FLIM-ML method for cervical cancer prediction were 90.9% and 100%, respectively. Compared with the LBC test currently used in clinical practice, the specificity and sensitivity of the FLIM-ML method are significantly higher. In the follow-up cancer care group, one recurrence case was predicted to be high risk by FLIM-ML eight months earlier than the clinical methods. The FLIM-ML method is expected to have great application potential as a noninvasive, sensitive, and rapid screening method for cervical cancer and a convenient tool for follow-up cancer care.

## Figures and Tables

**Table 1:** Distribution of the 71 participants in the training dataset and the validation dataset based on their clinical diagnosis.

Clinical Diagnosis	Training Dataset	Validation Dataset
Cervical cancer	5	6
CINII/III	4	3
Benign	0	18
Normal	14	9
Follow-up	0	12
Total number	23	48

**Table 2:** Results of clustering of cell images in the training dataset.

Input Images	Group	Cluster 1	Cluster 2
*t_m_* images	CC/CINII-III	114/151 (75.5%)	37/151 (24.5%)
	Normal	5/217 (2.3%)	212/217 (97.7%)
*a*_2_ images	CC/CINII-III	114/151 (75.5%)	37/151 (24.5%)
	Normal	0/217 (0%)	217/217 (100%)
*t_m_* & *a*_2_ images	CC/CINII-III	114/151 (75.5%)	37/151 (24.5%)
	Normal	0/217 (0%)	217/217 (100%)

**Table 3:** Percentage of abnormal images in the validation group and the results of FLIM combined with machine learning. For comparison, the results of the LBC test of the patients are also listed.

Patient No.	Percentage of Abnormal Images			FLIM-ML	LBC Test
	*t_m_* Images	*a*_2_ Images	*t_m_* & *a*_2_ Images		
CC-2 (stage IB3)	100.0	100.0	100.0	+	+
CC-4 (stage IB2)	100.0	100.0	95.6	+	+
CC-6 (stage IIB)	100.0	100.0	100.0	+	+
CC-8 (stage IA1)	2.5	0.0	0.0	−(FN)	+
CC-10 (stage IIA1)	100.0	100.0	100.0	+	+
CC-11 (stage IIB)	73.7	100.0	89.5	+	+
CINII-2	83.3	83.3	58.3	+	−(FN)
CINII-4	50.0	80.0	50.0	+	+
CINII-6	78.3	91.3	78.3	+	+
Benign-1	0.0	0.0	0.0	−	−
Benign-2	0.0	0.0	0.0	−	−
Benign-3	8.3	8.3	8.3	−	−
Benign-4	4.5	0.0	0.0	−	−
Benign-5	0.0	0.0	0.0	−	−
Benign-6	0.0	0.0	0.0	−	−
Benign-7	45.5	0.0	0.0	−	−
Benign-8	73.3	40.0	40.0	−	−
Benign-9	0.0	0.0	0.0	−	−
Benign-10	0.0	0.0	0.0	−	−
Benign-11	36.4	9.1	9.1	−	−
Benign-12	0.0	0.0	0.0	−	−
Benign-13	0.0	11.1	0.0	−	−
Benign-14	20.0	20.0	20.0	−	−
Benign-15	54.5	9.1	0.0	−	−
Benign-16	0.0	0.0	0.0	−	−
Benign-17	36.4	45.5	36.4	−	−
Benign-18	58.3	0.0	0.0	−	+(FP)
Normal-15	0.0	0.0	0.0	−	−
Normal-16	0.0	0.0	0.0	−	−
Normal-17	0.0	0.0	0.0	−	−
Normal-18	0.0	0.0	0.0	−	−
Normal-19	50.0	0.0	15.4	−	+(FP)
Normal-20	0.0	23.1	0.0	−	+(FP)
Normal-21	36.4	0.0	0.0	−	+(FP)
Normal-22	0.0	0.0	0.0	−	+(FP)
Normal-23	0.0	0.0	0.0	−	−
Follow-up-1	10.0	0.0	0.0	−	−
Follow-up-2	0.0	0.0	0.0	−	−
Follow-up-3	10.0	0.0	0.0	−	−
Follow-up-4	13.3	13.3	13.3	−	−
Follow-up-5 (VINII-III)	85.0	100.0	70.0	+	+
Follow-up-6	0.0	37.5	0.0	−	−
Follow-up-7 (VAINIII)	100.0	88.0	76.0	+	−(FN)
Follow-up-8	5.0	55.0	10.0	−	−
Follow-up-9	0.0	45.0	10.0	−	−
Follow-up-10	5.0	0.0	0.0	−	−
Follow-up-11	15.0	40.0	15.0	−	−
Follow-up-12	5.0	5.0	0.0	−	−

FP: false positive; FN: false negative.

**Table 4:** Sensitivity and specificity of the LBC test and FLIM-ML method.

Method	Sensitivity (%)	Specificity (%)
LBC	81.8	86.5
FLIM-ML	90.9	100

## Data Availability

All data supporting the findings of this study are available from the corresponding author upon reasonable request.

## Supplementary Materials

The supporting information can be downloaded at: https://www.mdpi.com/article/10.3390/ijms231911476/s1.
