# Supplementary material for: Early Detection of Cervical Cancer by Fluorescence Lifetime Imaging Microscopy Combined with Unsupervised Machine Learning
Source: Int J Mol Sci. 2022 Sep 29;23(19):11476. doi: 10.3390/ijms231911476 (PMC9570424; doi:10.3390/ijms231911476)
Supplement: Supplementary file 1 [file ijms-23-11476-s001.zip › ijms-1899293-supplementary.pdf]

International Journal of *Molecular Sciences*

MDPI

Article

Early detection of cervical cancer by fluorescence lifetime imaging microscopy combined with unsupervised machine learning

Mingmei Ji [1,†], Jiahui Zhong [2,†], Runzhe Xue [1], Wenhua Su [1], Yawei Kong [1], Yiyan Fei [1], Jiong Ma [1,2,3], Yulan Wang [4,*], and Lan Mi [1,2,*]

[1] Department of Optical Science and Engineering, Shanghai Engineering Research Center of Ultra-precision Optical Manufacturing, Key Laboratory of Micro and Nano Photonic Structures (Ministry of Education), School of Information Science and Technology, Fudan University, 220 Handan Road, Shanghai 200433, China
[2] Institute of Biomedical Engineering and Technology, Academy for Engineering and Technology, Fudan University, Shanghai 200433, China
[3] Shanghai Engineering Research Center of Industrial Microorganisms, The Multiscale Research Institute of Complex Systems (MRICS), School of Life Sciences, Fudan University, 220 Handan Road, Shanghai 200433, China
[4] Department of Gynecology and Obstetrics, The Central Hospital of Wuhan, Tongji Medical College, Huazhong University of Science and Technology, 26 Shengli Street, Wuhan 430014, China
* Correspondence: wangyulan@zxhospital.com (Y.W); lanmi@fudan.edu.cn (L.M.)
† These authors contributed equally to this work.

Supplementary Materials

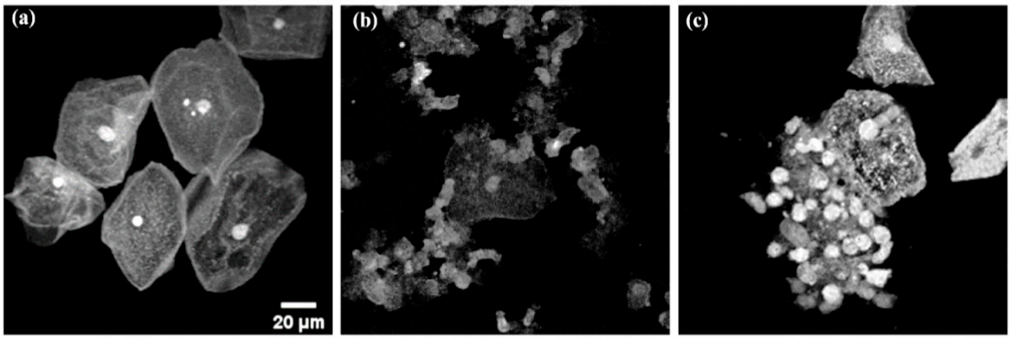

Figure S1. Typical qualified and unqualified images. (**a**) A qualified image showing cervical exfoliated cells. (**b**) An unqualified image, in which the fluorescence intensity of exfoliated cells was much lower than other area. (**c**) Another unqualified image with many neutrophils covering the cervical exfoliated cells.

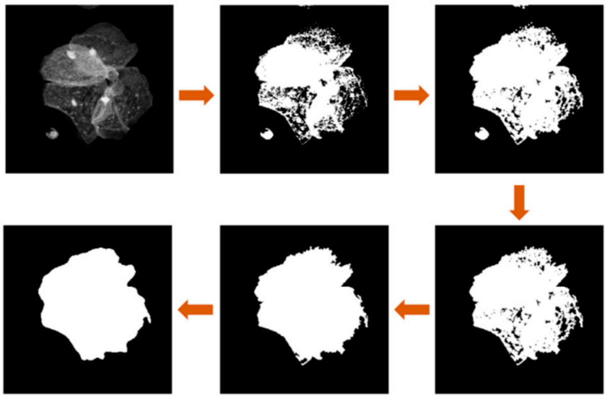

Figure S2. Diagram of an automatic segmentation algorithm for the cervical exfoliated cells mask region process.
